# The prognostic role of PD-L1 expression and the presence of polyomavirus in Merkel cell carcinoma cases

**DOI:** 10.1186/s13027-023-00564-1

**Published:** 2024-01-04

**Authors:** Stella Meireles Siqueira, Gabriella Campos-do-Carmo, Paulo Ricardo Garcia da Silva, Isabele Ávila Small, Andreia Cristina De Melo

**Affiliations:** 1 Division of Clinical Research and Technological Development, Brazilian National Cancer Institute, Rio de Janeiro, Brazil; 2 Dermatology, Gávea Medical Center, Rio de Janeiro, Brazil; 3 Division of Pathology, Brazilian National Cancer Institute, Rio de Janeiro, Brazil

**Keywords:** Merkel cell carcinoma, Merkel cell polyomavirus, Programmed death ligand-1, Skin cancer

## Abstract

**Background:**

Merkel cell carcinoma (MCC) comprises a rare malignant primary skin tumor presenting neuroendocrine differentiation. Recently, agents blocking the programmed cell death protein 1 and programmed cell death protein ligand 1 pathway (PD-1/PD-L1) have demonstrated objective and durable tumor regressions in patients presenting advanced MCC. This study aimed to describe the sociodemographic, clinical, and histopathological characteristics of MCC patients, also assessing the prevalence of PD-L1 expression and Merkel cell Polyomavirus (MCPyV), as well as their prognostic roles.

**Methods:**

Data from patients diagnosed with MCC between 1996 and 2019 at a reference cancer center in Rio de Janeiro, southeastern Brazil, were evaluated in a retrospective study. Tumor samples were tested for MCPyV and PD-L1 employing immunohistochemistry. Survival analyses were carried out employing the Kaplan–Meier method and curves were compared using the log-rank test. A multiple semiparametric Cox model was used. Values *p* < 0.05 were considered significant.

**Results:**

A total of 65 patients were included in the study, with a mean age at diagnosis of 72 (standard deviation 13.9). A total of 56.9% (37/65) of the patients were male, 86.2% (56/65) were white, and 56.9% (37/64) were illiterate or with incomplete elementary school. MCPyV immunohistochemistry was positive in 29 cases (44.6%) and PD-L1 positivity was ≥ 1% in 42 cases (64.6%). Significant associations between MCPyV and PD-L1 expression ≥ 1% (*p* = 0.003) and PD-L1 expression ≥ 5% (*p* = 0.005) were noted. Concerning the multivariate analysis, only education level and advanced MCC stage indicated statistically significant worse progression-free survival. Regarding overall survival (OS), being male, education level and advanced stage comprised risk factors. The estimated OS at 60 months for stages I to III was of 48.9% and for stage IV, 8.9%.

**Conclusions:**

This is the first large Brazilian cohort to assess the prevalence of MCPyV in MCC tumors, as well as PD-L1 expression and their associations. No correlations were noted between MCPyV infection or PD-L1 expression and survival rates.

## Background

Merkel cell carcinoma (MCC) is a rare and aggressive type of skin cancer of neuroendocrine origin [1], first described in 1972 by Toker as trabecular skin carcinoma [2]. Since then, knowledge concerning the pathophysiology of this condition and patient management has advanced exponentially. Also known as primary cutaneous neuroendocrine carcinoma, MCC received this denomination due to its ultrastructural and immunophenotypic similarity to Merkel sensory skin cells [2]. However, the Merkel cell origin for MCC was considered unlikely as this carcinoma can actually derive from a neuronal precursor [3–5].

In 2008, Feng et al. described the association between a new polyomavirus and MCC for the first time, later named Merkel cell polyomavirus (MCPyV) [6]. The prevalence of subclinical MCPyV infections, reported as around 60 to 80% in adults, increases with age. This may, however, differ significantly among different geographic regions, such as in Australia, where a much lower association with this viral infection, of around 25%, has been noted [7].

Two distinct MCC etiologies have been described: clonal integration of MCPyV DNA into tumor genomes and UV damage. Both forms exhibit high proliferative growth rates due to mutations in oncogenes and tumor suppressor genes. These include the RB, p53, MYCL, and ATOH1 pathways [8]. Furthermore, emerging evidence indicates that dysregulation of epigenetic mechanisms, including histone post-translational modifications, are involved in MCC. Furthermore, histone acetylation, methylation and phosphorylation mark impairments, as well as histone modifying enzymes, have also been implicated in this disease [9].

Still concerning MCC causes, chronic immunosuppression, including use of immunosuppressive drugs, HIV/AIDS, lymphoproliferative disorders, solid organ transplantation and auto-immune diseases have all been associated with increased risk of developing MCC. Compared to immune-competent MCC patients, immunosuppressed MCC patients display significantly worse MCC-specific and overall survival [10].

Results are variable regarding the prognostic role of MCPyV status, with predominantly worse prognoses observed in patients presenting polyomavirus-negative tumors or with no difference concerning MCPyV-positive tumors [11]. Both MCPyV positive and negative patients can, however, present clinically aggressive and fatal courses [7].

MCC is characteristically aggressive and locally invasive, with high local recurrence rates and the involvement of regional lymph nodes [1]. A recent study reported a 40% recurrence rate at 5 years for this condition, with a first year risk of recurrence of 11% for stage I patients, 33% for stage IIA/IIB, 30% for stage IIIA, 45% for stage IIIB, and 58% for stage IV, with 95% of all recurrences ensuing in the first 3 years. Other risk factors associated with increased recurrence rates comprise immunosuppression, being male, clinically detectable nodal disease, and advanced age [12].

The limited number of therapeutic MCC treatment options leads to an urgent need to determine tumor-specific pathways and possible therapeutic targets [1]. In this sense, an increasing body of evidence on the role of the immune system in MCC control has paved the way for the use of checkpoint inhibitors, namely anti-PD-1 (programmed cell death 1) and anti-PD-L1 (programmed cell death protein ligand 1) [13].

This study, therefore, aimed to assess clinical MCC patient characteristics, the prevalence of MCPyV and PD-L1 expression, as well as the prognostic role of these biomarkers.

## Methods

### Study design, patient selection, and data collection

This study was approved by the Brazilian National Cancer Institute (INCA) Ethics in Human Research Committee and conducted following Good Clinical Practice guidelines.

INCA patients were included when older than 18 years old and wen presenting a confirmed histopathological MCC diagnosis between 1996 and 2019. Exclusion criteria comprised patients whose records lacked the clinical data of interest and paraffin-embedded tumor samples.

Patient characteristics, comprising age at diagnosis, sex, phototype, comorbidities, staging, related treatments, response, and survival information, were retrospectively obtained from hospital records and entered into the Research Data Capture (REDCap®) system. All cases were reclassified following the American Joint Committee on Cancer AJCC (AJCC) Cancer Staging Manual 8th edition [14].

### Histopathological and immunohistochemistry evaluations

A trained pathologist reviewed all specimens concerning the following morphological variables: mitotic index, fibroplasia, inflammatory infiltrate, tumor thickness, ulceration, necrosis, hemorrhage and subtype [15]. Primary tumor samples were preferentially used. If not available, metastases were analyzed.

MCPyV (clone CM2B4, Santa Cruz, 200 µg/mL dilution, 0.1 mL) expression was classified as positive when tumor cells exhibited a dark staining. PD-L1 (clone SP263, Ventana) was quantified based on the percentage of positive cells in 10 fields at 10 magnifications over the total cell number. Tumor cell staining was compared with positive and negative controls. Two cutoff points were employed for the PD-L1 evaluations, namely < 1% (negative) and ≥ 1% (positive) or < 5% (negative) and ≥ 5% (positive).

### Statistical analyses

Statistical analyses were performed in the R environment [16]. A descriptive analysis of the investigated variables was performed. Means and standard deviations (SD) (or medians and interquartile ranges [IQR], when applicable) were presented for continuous variables. Categorical variables were described by their absolute and relative frequencies and missing data were excluded from the analyses.

Pearson's chi-square test was applied to assess the association between MCPyV and PD-L1.

Outcomes were evaluated from the date of the first histopathological report confirming the MCC diagnosis and the date of the first recurrence/disease progression or death for progression-free survival (PFS), and patients presenting no recurrence/progression or who died were censored on the date of the last contact. Concerning overall survival (OS), the interval between the histopathological MCC diagnosis date and the date of death from any cause was calculated, and patients alive or lost during follow-up were censored on the date of the last contact. The Kaplan–Meier method was employed to estimate the PFS and OS medians and the curves were compared by the log-rank test [17]. The Hazard Ratios (HR) and the respective confidence intervals (95%CI) for each risk factor were obtained by the semiparametric Cox model [18]. Variables were manually included for multiple model adjustment, according to *p* < 0.10 values obtained by the univariate Cox model. A *p*-value of < 0.05 was considered as statistically significant [18].

## Results

A total of 65 patients were included in the study, 37 males (56.9%) and 28 females (43.1%). Patient baseline demographic, clinical and pathological characteristics are described in Table 1. Mean age at diagnosis was of 72 years old (SD 13.9). Patients were predominantly white (86.2%), with 56.9% (37/64) illiterate or who had not completed elementary school and 41.5% (27/64) completing elementary school or above.

**Table 1:** Baseline demographic, clinical and pathological characteristics

	n (%)
*Age (years)*	
Mean (SD)	72 (13.9)
Median (IQR)	73 (19)
*Sex*	
Male	37 (56.9)
Female	28 (43.1)
*Race*	
White	56 (86.2)
Other	9 (13.8)
*Education*	
Illiterate or incomplete elementary school	37 (56.9)
Complete elementary school or above	27 (41.5)
Missing	1 (1.6)
*Primary tumor site*	
Head and neck	25 (38.5)
Lower limbs	21 (32.3)
Upper limbs	10 (15.4)
Trunk	9 (13.8)
*Clinical diameter of the primary tumor in millimeters*	
Median (IQR)	40 (45.5)
*Disease stage*	
I–II	31 (47.7)
III	13 (20.0)
IV	14 (21.5)
Missing	7 (10.8)
*Comorbidities*	
Hypertension	33 (50.8)
Diabetes mellitus	15 (23.1)
Chronic renal failure	4 (6.2)
HIV	2 (3.1)
Lymphoproliferative neoplasia	1 (1.5)
*Initial treatment*	
Surgery	44 (67.7)
Chemotherapy	8 (12.3)
Radiotherapy	17 (26.2)
Immunotherapy	0 (0)
*General histopathological aspects*	
Mitotic index—Median (IQR)	24/10HPF (37)
Fibroplasia present	59 (90.8)
Tumor thickness in millimetres—Mean (SD)	17.9 (26.5)
Ulceration present	20 (30.8)
Necrosis present	22 (33.8)
Hemorrhage present	31 (47.7)
*Histological subtype*	
Nodular	40 (61.5)
Infiltrative	24 (36.9)
Missing	1 (1.6)

HPF: high power field. IQR: SD: standard deviation. IQR: Interquartile range

Comorbidities were observed in 41 patients, with systemic arterial hypertension present in 50.8% (33/65) of cases, diabetes mellitus in 23.1% (15/65), chronic renal failure in 6.2% (4/65), HIV infection in 3.1% (2/65) and lymphoproliferative neoplasia in 1.5% (1/65).

The most common primary tumor site was the head and neck region (38.5%), followed by the lower limbs (32.3%), upper limbs (15.4%) and trunk (13.8%). Concerning the clinical primary tumor diameter, the minimum reported size was of 4 mm and the maximum, 150 mm, with a median of 40 mm (IQR 45.5). According to the AJCC 8th edition, 47.7% (31/58) of the cases were classified as stage I or II, 20.0% (13/58) as stage III and 21.5% (14/58) as stage IV.

The mitotic index median was 24/10 high power field (IQR 37). Fibroplasia was present in 90.8% (59/65) of the cases. Mean tumor thickness was 17.9 mm (minimum 1.8 mm and maximum 160 mm). Ulcerations were present in 30.8% (20/65) of the patients, necroses in 33.8% (22/65) and hemorrhages, in 47.7% (31/65). The most frequent subtype was nodular, in 61.5% (40/64) of the cases, while the infiltrative subtype was observed in 36.9% (24/64).

**Fig. 1:**
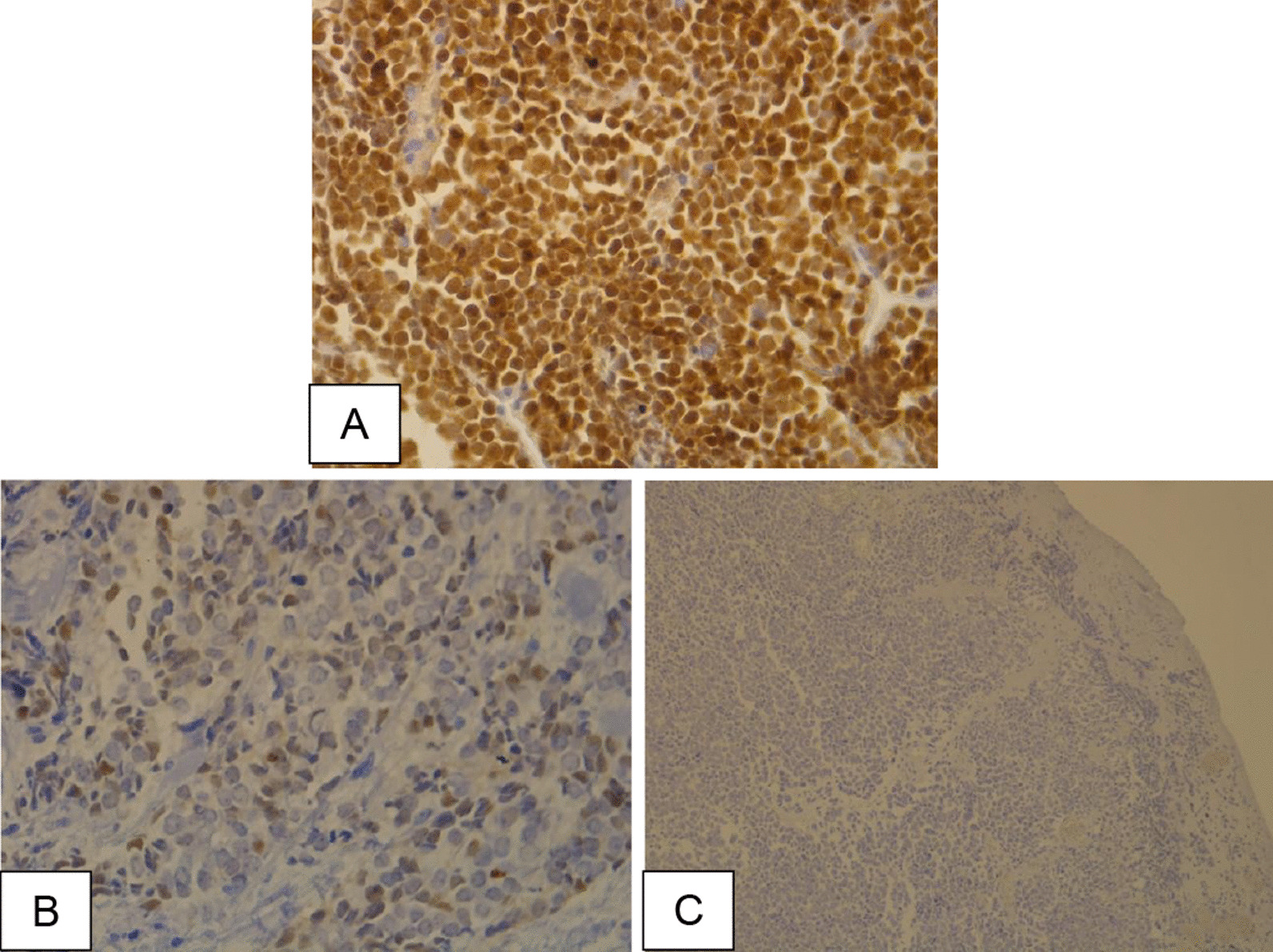
Immunohistochemistry **A** positive control for MCPyV (40 × magnification). **B** MCPyV-positive case (40 × magnification). C) MCPyV-negative case (4 × magnification)

**Fig. 2:**
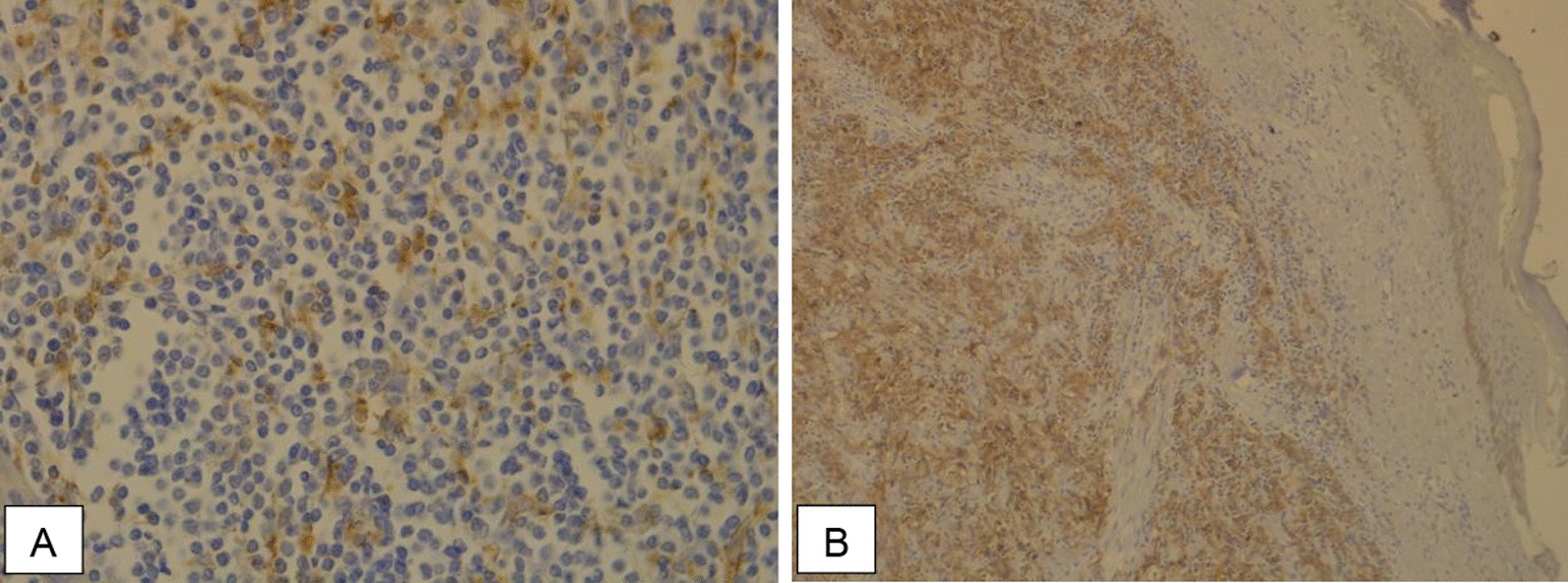
Immunohistochemistry **A** PD-L1-positive control (40 × magnification). **B** PD-L1-positive case—80% positivity with a diffuse distribution pattern (10 × magnification)

A total of 29 cases were positive for MCPyV (44.6%) (Fig. 1). PD-L1 expression was ≥ 1% in 42 cases (64.6%) and ≥ 5% in 17 cases (26.2%). Among the 42 patients in which PD-L1 was different from 0%, a focal distribution pattern was observed in 17 cases (40.5%), multifocal in 16 cases (38.1%), diffuse in eight cases (19.0%) and undetermined in one (2.4%) (Fig. 2). Significant associations between MCPyV and PD-L1 expression ≥ 1% (*p* = 0.003) and PD-L1 expression ≥ 5% (*p* = 0.005) were noted (Tables 2 and 3).

**Table 2:** Pearson's chi-square test with continuity correction (Yates correction) comparing the dichotomous variable PD-L1 (1%) by MCPyV status

	PD-L1 < 1%	PD-L1 ≥ 1%	*p*-value
Negative MCPyV	19 (52.8%)	17 (47.2%)	0.003
Positive MCPyV	4 (13.8%)	25 (86.2%)	
Total	23	42

**Table 3:** Pearson's chi-square test with continuity correction (Yates correction) comparing the dichotomous variable PD-L1 (5%) by MCPyV status

	PD-L1 < 5%	PD-L1 ≥ 5%	*p*-value
Negative MCPyV	32 (88.9%)	4 (11.1%)	0.005
Positive MCPyV	16 (55.2%)	13 (44.8%)	
Total	48	17	

The median follow-up period was of 98.6 months. The estimated PFS at 60 months and 120 months for stages I, II and III were 42.6% (95%CI 30.1–60.3%) and 18% (95%CI 6.6–48.7%), respectively.

Absolute number of deaths comprised 49 cases, 43 due to MCC and six from other causes, namely hemorrhagic stroke, acute myocardial infarction, prostate cancer, skin squamous cell carcinoma, COVID-19, and one external cause.

Concerning the univariate analysis, being male (HR = 2.15, 95%CI 1.11 to 4.15, *p* = 0.023), with a low education level (HR = 2.68, 95%CI 1.40–5.11, *p* = 0.003) and presenting an advanced MCC stage (HR = 4.11, 95%CI 1.95–8.68, *p* < 0.001) were associated with worse PFS. In the multivariate analysis, only education level (HR = 2.29, 95%CI 1.19–4.41, *p* = 0.013) and advanced stage (HR = 3.36, 95%CI 1.58–7.18, *p* = 0.002) led to statistically significant worse PFS (Table 4).

**Table 4:** Univariate and multivariate analyses for PFS

	n	HR (univariate)	HR (multivariate)
*Sex*			
Female	23	–	–
Male	35	2.15 (1.11–4.15, *p* = 0.023)	
*Education level*			
Complete elementary school or above	26	–	–
Illiterate or incomplete elementary school	32	2.68 (1.40–5.11, *p* = 0.003)	2.29 (1.19–4.41, *p* = 0.013)
*Disease stage*			
I–III	44	–	–
IV	14	4.11 (1.95–8.68, *p* < 0.001)	3.36 (1.58–7.18, *p* = 0.002)
*Surgery (initial treatment)*			
No	19	–	–
Yes	39	0.25 (0.13–0.47, *p* < 0.001)	
*MCPyV*			
Negative	31	–	–
Positive	27	0.67 (0.36–1.25, *p* = 0.211)	
*PD-L1 expression*			
< 1%	21	–	–
≥ 1%	37	0.79 (0.43–1.47, *p* = 0.461)	

HRs and respective 95%CIs estimated by the Cox model for the semiparametric PFS outcome

The estimated OS at 60 months and 120 months for stages I, II and III were 48.9% (95%CI 35.9–66.2%) and 17.8% (95%CI 6.5–48.5%), respectively, and 8.9% for stage IV only at 60 months (95%CI 1.4–56%).

**Fig. 3:**
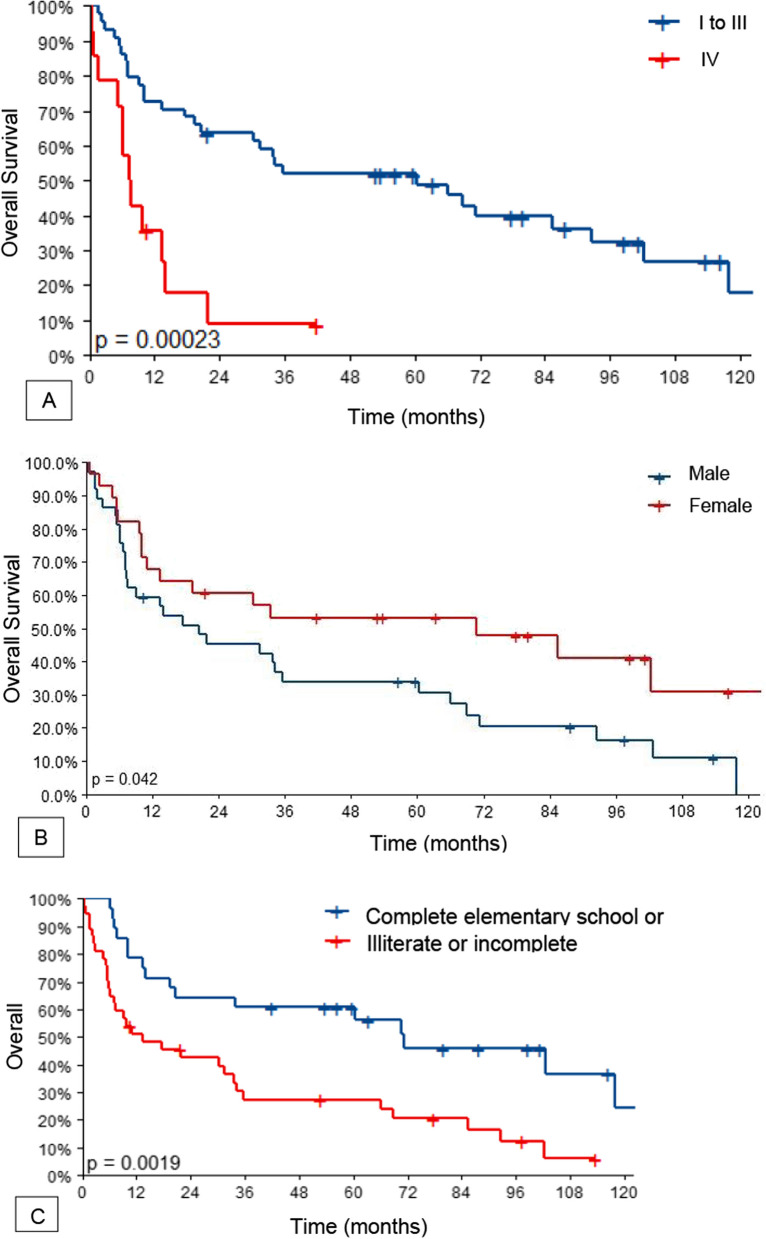
**A** Overall Survival Curve according to AJCC staging 8th edition. **B** Overall Survival Curve according to sex. **C** Overall Survival Curve according to education level

Being male, education level and advanced MCC stage were associated with worse OS in both the univariate and multivariate analysis. In the multivariate analysis, being male (HR = 2.30, 95%CI 1.14–4.67, *p* = 0.021), education level (HR = 2.13, 95%CI = 1.09–4.19, *p* = 0.028) and advanced MCC stage (HR = 3.31, 95%CI 1.54–7.09, *p* = 0.002) led to statistically significant worse OS (Table 5). The OS curves according to the stage, sex and education level are depicted in Fig. 3.

**Table 5:** Univariate and multivariate analyses for OS

	n	HR (univariate)	HR (multivariate)
*Sex*			
Female	23	–	–
Male	35	2.67 (1.33–5.38, *p* = 0.006)	2.30 (1.14–4.67, *p* = 0.021)
*Education level*			
Complete elementary school or above	26	–	–
Illiterate or incomplete elementary school	32	2.59 (1.33–5.03, *p* = 0.005)	2.13 (1.09–4.19, *p* = 0.028)
*Disease stage*			
I–III	44	–	–
IV	14	3.72 (1.77–7.81, *p* = 0.001)	3.31 (1.54–7.09, *p* = 0.002)
*Surgery (initial treatment)*			
No	19	–	–
Yes	39	0.25 (0.13–0.49, *p* < 0.001)	
*MCPyV*			
Negative	31	–	–
Positive	27	0.70 (0.37–1.33, *p* = 0.278)	
*PD-L1 expression*			
< 1%	21		–
≥ 1%	37	0.84 (0.45–1.59, *p* = 0.598)	

HRs and respective 95%CIs estimated by the Cox model for the semiparametric OS outcome

MCPyV was not statistically correlated with PFS (HR = 0.67, 95%CI 0.36–1.25, *p* = 0.211) or OS (HR = 0.70, 95%CI 0.37–1.33, *p* = 0.278). Likewise, PD-L1 expression was not statistically correlated with PFS (HR = 0.79, 95%CI 0.43–1.47, *p* = 0.461) or OS (HR = 0.84, 95%CI 0.45–1.59, *p* = 0.598) (Table 5).

## Discussion

MCC rates are usually higher in men (62.1%) compared to women (37.9%) considering most available epidemiological data [14]. In Brazil, Melo et al. published a study on 881 MCC patients reporting a slight female predominance (51.2%) [19]. MCC occurs more frequently in patients over 60 [14, 19, 20]. Herein, MCC was noted as more frequent in male patients, with a mean age of 72, similar to that described in the literature. A white ethnicity is reported as predominant in the literature [14, 19], also noted herein.

Being older and male are associated with worse OS according to the literature [21, 22], although age was not a prognostic factor in the present study.

Herein, low education levels (illiterate or incomplete elementary school) comprised a risk factor concerning PFS and OS in the multivariate analysis. Data correlating schooling and survival in MCC cases, however, are not yet been available. Conversely, correlations between low education and worse survival have been described for other malignant tumors [23, 24]. Low education levels can be associated with lower treatment adherence, greater difficulty in accessing health services, and consequently, late diagnoses, all of which negatively influence case management and outcomes, potentially explaining the obtained results.

According to the National Cancer Database (NCDB) data, MCC is most common in the head and neck region (42.6%), followed by upper limbs and shoulders (23.6%) [14]. Andea et al. reported the extremities as the most common primary tumor site (42%), followed by the head and neck (37%) [25]. In the present study, the most common topography was head and neck, curiously followed by the lower limbs.

Average clinical MCC tumor sizes in the literature range from 7 to 30 mm [26–30]. Herein, however, the median tumor diameter was larger than published data. Similarly, mean tumor thickness reported in the literature ranges from 8.8 to 12.3 mm [25, 31], with the mean thickness observed in this cohort also larger than literature data. This may be due to delays in accessing the tertiary site, resulting in large and profound primary tumors at the time of hospital admission.

According to the literature, immunosuppression comprises an MCC risk factor, which is more common in transplanted patients, patients presenting lymphoproliferative neoplasms and HIV-infected patients [32, 33]. A low frequency of these comorbidities was, however, observed herein. Systemic arterial hypertension, on the other hand, was present in 50.8% of the cases and diabetes mellitus, in 23.1%, potentially due to the confounding factor of advanced age, as these comorbidities are more common in elderly patients.

Some studies have not reported correlations between MCC thickness and OS [29, 31]. Andea et al. however, in their study on 156 patients, reported that thicker tumors were associated with decreased survival rates [25]. Herein, tumor thickness alone was not correlated to PFS or OS.

Llombart et al. reported a predominant nodular tumor growth pattern in 70% of their cases [29]. Mott et al. reported that an infiltrative growth pattern is associated with adverse prognosis [30], while two studies have demonstrated that a nodular pattern is related to better survival rates [25, 34].

Melo et al., in a study conducted in Brazil, reported that MCC diagnosis occurs predominantly at stages III or IV (50.5%) [19]. Herein, most patients were diagnosed at stages I and II.

According to the AJCC Cancer Staging Manual 8th edition, 5-year OS estimates for clinical staging comprise 45.0% for stage I, 30.9% IIA and 27.3% for stage IIB, respectively. The 5-year OS for the revised stage IIIA was 40.3% and 26.8% for stage IIIB [14]. Considering stage IV, the 2-year survival rate is of only 26% [1]. In the present study, the estimated 5-year OS for stage IV was only lower than the AJCC estimates, probably due to the previously mentioned service access issues and the lack of checkpoint inhibitors for systemic treatment.

The frequency of MCPyV detection depends on the applied method. Busam et al. reported 88% MCPyV positivity using polymerase chain reaction (PCR), decreasing to 67% when applying immunohistochemistry [35]. Similarly, Leroux-Kozal et al. reported 88% and 58% MCPyV positivity when employing PCR and immunohistochemistry, respectively [36]. Paik et al. reported that only 19 out of 104 (18.3%) Australian MCC cases exhibited positive immunohistochemical staining for MCPyV [37]. Another study concerning 37 cases using PCR suggested that MCPyV prevalence may be as low as 24% for Australian patients. The authors postulate that this may be explained due to greater ultraviolet exposure in Australia, resulting in MCC carcinogenesis uncontrolled by MCPyV [38]. Herein, almost half of the cases were MCPyV positive as determined by immunohistochemistry.

Several studies support a more favorable prognosis of MCPyV-positive MCCs. Moshiri et al., for example, in a study involving 282 cases, reported a better prognosis in MCPyV-positive cases. In another study, a multivariate analysis including age, sex and immunosuppression, indicated that patients with virus-negative MCC exhibited a significantly increased risk of disease progression (HR = 1.77, 95%CI 1.20–2.62) and death (HR = 1.85, 95%CI 1.19–2.89) [39]. Furthermore, Sihto et al., reported that MCPyV-positive cases display a higher survival rate compared to MCPyV-negative counterparts, presenting a 5-year survival of 45% versus 13% (*p* < 0.01), respectively [40]. Comparing this to the data obtained in the current cohort, MCPyV positivity exhibited a trend towards a protective exposure profile, albeit non-statistically significant, probably due to the small sample size.

Lipson et al. observed PD-L1 positivity in 49% of 67 MCC samples, considering a cutoff point of 5%. These authors also reported a statistically significant association between tumor cell PD-L1 expression and longer survival times (HR = 3.12, 95%CI 1.28–7.61, *p* = 0.012) [41]. Herein, the number of PD-L1-positive cases was lower when the cutoff was set at 5%. No statistically significant association was detected in the univariate analysis concerning the OS and PFS analysis, even when considering a cut-off point of 1% for PD-L1 positivity.

A significant association has been noted between MCPyV and PD-L1 in MCC tumors [41], with other studies observing similar results [42–44]. In this regard, MCPyV infection seems to promote the expression of immune response-associated proteins [42]. In the present study, a significant association between MCPyV and PD-L1 expression was observed.

Some limitations should be acknowledged concerning this assessment. First, the fact that this is retrospective study, which makes data collection difficult. It is also a single-center study encompassing a limited sample size, posing difficulties in conducting robust statistical analyses and increasing the margin of error. The rarity of the disease is also noteworthy, hindering cohort studies and leading to prolonged intervals concerning case selections. Moreover, during this period, the review and updating of MCC treatment protocols during the study period make it challenging to verify treatment efficacy. Finally, a considerable limitation arises from the unavailability if immunotherapy at the Brazilian National Cancer Institute, where the research was conducted, which may potentially impact survival data when compared to other institutions that offer this contemporary treatment.

## Conclusions

This is the first large Brazilian cohort to assess the prevalence of MCPyV in tumors, as well as PD-L1 expression and the association between these markers. The prevalence of MCPyV infection in MCC cases in the studied population was of 44.6%, although no statistically significant correlations with PFS and OS were observed. PD-L1 expression was ≥ 1% in 42 cases (64.6%) and ≥ 5% in 17 cases (26.2%). No statistical significance concerning PD-L1 correlations to PFS and OS were, however, noted. Significant associations between MCPyV and PD-L1 expression ≥ 1% (*p* = 0.003) and PD-L1 expression ≥ 5% (*p* = 0.005) were detected.

## Data Availability

The datasets used and/or analysed during the current study are available from the corresponding author on reasonable request.

## Abbreviations

AJCC: American Joint Committee on Cancer
CI: Confidence interval
HR: Hazard ratios
INCA: Brazilian National Cancer Institute
IQR: Interquartile range
MCC: Merkel cell carcinoma
MCPyV: Merkel cell polyomavirus
NCDB: National Cancer Database
OS: Overall survival
PCR: Polymerase chain reaction
PD-1: Programmed cell death 1
PD-L1: Programmed cell death protein ligand 1
PFS: Progression-free survival
REDCap: Research data capture
SD: Standard deviation
